# Effects of secukinumab on skeletal microarchitecture and vertebral fractures in patients with axial spondyloarthritis using HR-pQCT

**DOI:** 10.1007/s11657-025-01565-w

**Published:** 2025-06-07

**Authors:** Chloe Heiting, Don McMahon, Douglas N. Mintz, Linda Russell, Dalit Ashany, Weijia Yuan, Insa Mannstadt, Emily M. Stein, Susan M. Goodman

**Affiliations:** 1 Division of Rheumatology, Department of Medicine, Hospital for Special Surgery, 535 E 70 Th Street, New York, NY 10021 USA; 2 Division of Endocrinology, Department of Medicine, Hospital for Special Surgery, New York, NY USA; 3 Department of Radiology and Imaging, Hospital for Special Surgery, New York, NY USA

**Keywords:** Ankylosing spondylitis, Spondyloarthropathy, X-ray densitometry, Skeletal microarchitecture, HR-pQCT

## Abstract

***Summary*:**

Participants with active AxSpA beginning secukinumab with moderate disease activity had improvements in symptoms, but no substantial change in BMD and microarchitecture. Larger, longer-term controlled studies are needed to assess the impact of IL-17 blockade on skeletal health.

**Background:**

Axial Spondyloarthritis (AxSpA) is linked to poor skeletal health, but whether IL-17 blockade, effective for symptom improvement, improves skeletal health is unknown. We investigated the impact of secukinumab on skeletal features.

**Methods:**

We prospectively enrolled AxSpA patients beginning therapy with secukinumab and followed them for 24 months. Clinical assessments, serum bone turnover markers, and cervical and lumbar spine radiographs were obtained. Areal BMD (aBMD) and trabecular bone score (TBS) measured by dual energy x-ray absorptiometry (DXA, spine, hip, forearm), volumetric BMD (vBMD), and microarchitecture measured by high-resolution peripheral quantitative computed tomography (HR-pQCT, Xtreme CT2, at the tibia and radius) were performed annually. DXA and HR-pQCT assessments were compared to reference cohorts of sex- and age-matched individuals. Changes were assessed through Wilcoxon and paired *t*-tests.

**Results:**

Thirty AxSpA participants were enrolled, 50% female (13% postmenopausal) and 47% HLA-B27 positive. Mean symptom duration was 12 years, with moderate activity (BASDAI mean 5 [SD = 2]). Baseline DXA and HR-pQCT Z-scores were within 1 standard deviation of sex- and age-matched controls. BASDAI (− 33%, *p* < 0.01) and BASMI (− 22%, *p* = 0.01) improved, but there was no improvement in aBMD, TBS, or microarchitecture. By HR-pQCT, vBMD decreased (− 0.6%, *p* = 0.04), and cortical porosity increased (8.3%, *p* = 0.03). New vertebral fractures were not observed.

**Supplementary Information:**

The online version contains supplementary material available at 10.1007/s11657-025-01565-w.

## Introduction

The prototypic feature of ankylosing spondylitis (AS), a subset of axial spondyloarthritis (AxSpA), is spine fusion due to new bone formation and syndesmophyte formation. Paradoxically, AxSpA patients simultaneously form and reabsorb bone, and osteoporosis and fractures are frequent complications. Osteoporosis of the spine in AxSpA is well recognized and is reported in 30–50% of cases, but there is less information about bone density or microarchitecture in peripheral bone sites [1]. Vertebral fractures are three times more frequent than nonvertebral fractures in patients with AS [2, 3], but the risk of fractures of the distal forearm, hip, and proximal humerus is also increased [4].

Despite the established risk of fractures in AxSpA, the assessment of bone quality in AxSpA patients is challenging [5]. Ossification of the juxtaspinal ligaments and syndesmophyte formation may result in misleadingly high areal bone density (aBMD) measurements using standard dual energy absorptiometry (DXA) [6, 7], the gold standard clinical tool for the assessment of bone health. High-resolution peripheral computed tomography (HR-pQCT), a three-dimensional imaging technique that enables highly sensitive in vivo assessment of peripheral vBMD and microarchitecture [8], may be more useful for skeletal assessment in patients with AxSpA. We previously utilized the latest HR-pQCT technology (XtremeCT II) to cross-sectionally investigate skeletal microarchitecture in AxSpA patients and relate bone quality to underlying disease features [9].

Treatment of AxSpA by cytokine blockade with either interleukin-17 (IL-17) blockade or tumor necrosis factor inhibitors (TNFi) improves pain and stiffness in patients with comparable efficacy. While both are recommended for AxSpA treatment [10, 11], their effects on skeletal health are less clear. In a post hoc analysis of data from a large clinical trial in patients with active AS, Braun et al. found an increase in BMD over 2 years of secukinumab treatment that was thought to be unrelated to the radiographic progression of syndesmophytes [12]. Studies by Haroon et al. have demonstrated improvements in BMD at the spine and hip over 1–3 years of treatment with TNFi in patients with AS [13], yet reduction in incident fractures or fracture severity with the use of TNFi therapy has not been demonstrated [3, 14]. We have previously reported that low bone density and abnormal microarchitecture in AxSpA were associated with disease duration and functional impairment using HR-pQCT [9]. Accordingly, the use of techniques such as HR-pQCT that overcome the limitations of standard assessments such as DXA may be more informative in the assessment of bone health in diseases such as AxSpA that have complex bone effects.

The purpose of this study was to investigate whether the initiation of IL-17 blockade with secukinumab improves bone turnover, BMD, and microarchitecture in AxSpA. We hypothesized that skeletal assessments would improve as clinical status improved with IL-17 blockade with secukinumab.

## Methods

Patients with AxSpA beginning therapy with secukinumab were enrolled between 2018 and 2021 in a prospective study investigating bone health and the effects of secukinumab therapy on bone. Patients with AxSpA who met the Assessment of Spondyloarthritis International Society (ASAS) Classification Criteria by X-ray (radiographic AxSpA or AS, defined as bilateral Grade 2 or unilateral Grade 3 or 4 changes) or had evidence of active sacroiliac inflammation on magnetic resonance imaging (MRI) but no or mild sacroiliac joint changes on radiographs (non-radiographic AxSpA) [15–17] were referred to the study or identified through the electronic medical record (EMR). Patients were excluded if they were pregnant, nursing, or planning to become pregnant within the subsequent two years, or if there was a history of fragility fracture, Cushing’s disease, primary hyperparathyroidism, multiple myeloma, osteomalacia, menopause or perimenopause, untreated vitamin D deficiency (25 OH D < 20 ng/ml), other forms of secondary osteoporosis, or other systemic rheumatic diseases. Patients were also excluded if they used oral steroids for two weeks in the six months prior to enrollment, used hormone replacement therapy at the time of screening, or had past or current use of intravenous (IV) bisphosphonate, teriparatide, denosumab, or romosozumab therapies. Patients with previous TNFi or other biologic therapy were enrolled after the appropriate washout period (4 weeks for etanercept, 8 weeks for infliximab, 10 weeks for adalimumab, 10 weeks for golimumab, 10 weeks for certolizumab, 6 months for ustekinumab). Patients were not using certolizumab or tofacitinib at the time of enrollment. Patients followed a standard of care dosing quantity and schedule, as prescribed by their treating rheumatologist: a loading dose of 150 mg or 300 mg subcutaneously at 0, 1, 2, 3, and 4 weeks after enrollment; and a prescribed dose of 150 mg or 300 mg subcutaneously every 4 weeks after the loading dose. 93.3% of patients were prescribed a dose of 150 mg. This protocol was approved by the Hospital for Special Surgery Institutional Review Board (IRB#2017–0660). Written informed consent was obtained from all participants included in the study.

### Assessment of clinical and disease characteristics

Surveys were completed by all participants to obtain demographics, information regarding calcium and vitamin D dietary intake and supplement use, and prior use of biologic medication (adalimumab, infliximab, golimumab, etanercept, ustekinumab, tocilizumab, and rituximab). Baseline, 2-month, and 1- and 2-year disease impact was assessed using standardized measurement tools including bath ankylosing spondylitis disease activity (BASDAI, score ≥ 4/10 considered “active”), functional index (BASFI, 0 = no functional impairment and 10 = severe functional impairment), metrology index (BASMI, 0 = no limitation of mobility and 10 = severe limitation), and Ankylosing Spondylitis Disease Activity Score with C-reactive protein (ASDAS-CRP, score of 2.1–3.5 indicates high disease activity and > 3.5 signifies very high disease activity) [18–21]. BASDAI, BASFI, and BASMI were assessed by experienced rheumatologists.

### Assessment of biomarkers

Serum for measurement of human leukocyte antigen (HLA) B27, erythrocyte sedimentation rate (ESR), CRP, and intact parathyroid hormone (iPTH) were obtained at the baseline visit. Bone turnover markers and calciotropic hormones were measured at baseline, 2 months, and 1 and 2 years, including bone formation measured as osteocalcin and bone specific alkaline phosphatase; bone resorption measured as C-telopeptide (CTX); and IL-17 and TNF-alpha levels.

### Imaging assessment

Imaging was obtained at baseline and 1 and 2 years and included DXA measures, HR-pQCT, and radiographs of the cervical and lumbar spine. aBMD was measured at the lumbar spine (L1-L4), total hip, femoral neck, and 1/3 radius using DXA (Horizon A [S/N 201056] densitometer, Hologic, Inc., Waltham, MA). The in vivo precision of the measurements at our institution is 0.70% for the spine, 1.36% for the total hip, and 0.70% for the radius. The Hologic healthy reference population of the same age and sex was used to determine Z-scores. HR-pQCT scans were taken of the non-dominant distal radius and tibia using the Xtreme II scanner (Scanco Medical, Brüttisellen, Switzerland) except in cases of a prior fracture at the site or metal implant, in which case the contralateral limb was scanned. The Xtreme II utilizes a microfocus X-ray source (68 kVp voltage, 900 µA current, 43-ms integration time) to scan a 10.2 mm area along the axis of the long bone, resulting in a 60.7-µm isotropic voxel size. The region of interest was defined by a two-dimensional scout film using a reference line from the distal end plate with a fixed offset of 9 mm for the radius and 22 mm for the tibia. All scans were performed and analyzed by a single trained operator who evaluated for motion with a score of 1–5 and excluded those scoring over 3. The images were filtered and binarized using the standard method of the manufacturer, and an automated segmentation algorithm was used to distinguish cortical and trabecular regions [22]. The HR-pQCT measures assessed included total (Tt), trabecular (Tb), and cortical (Ct) area (Ar), volumetric BMD (vBMD), thickness (Th), trabecular number (Tb.N), separation (Tb.Sp), and cortical porosity (Ct.Po). The in vivo short-term reproducibility of these measures at the center is between 0 and 5% for all measures except cortical porosity [8]. Z-scores for HR-pQCT measures were generated based on comparisons to age- and sex-matched healthy controls, using the XCTII Normative© data from a western Canadian cohort of 1236 people including 768 females and 468 males [23].

### Radiographic analysis

Cervical and lumbar spine radiographs were evaluated using the modified stoke Ankylosing Spondylitis Spine Score (m-SASSS) method, with a possible total score range of 0 to 72 [24]. Some spinal segments were not visible in all films, and images lacking more than three segments were excluded. Due to the absence of adequate cervical and lumbar x-ray films for each patient, the score was calculated as 12 times the mean score of all scoring sites, with a possible range of 0 to 36 [25]. Lateral spine radiographs were systematically assessed for the presence of vertebral fractures in all patients at baseline and 2 years. When available, DXA vertebral fracture assessment, MRIs, and CT scans were reviewed. A fracture was defined as at least 20% loss of height of a vertebra when compared to an adjacent vertebra [26].

### Statistical methods

All analyses were performed using SAS version 9.2 and SAS-STAT version 13.4. Descriptive statistics of patient demographics and clinical characteristics were performed, including mean and standard deviation (SD) for normally distributed continuous variables and proportions for categorical variables. T-tests and chi-squared tests were employed to determine differences in means and proportions between different genders Spearman correlations were used to relate patient characteristics and imaging features from DXA and HR-pQCT.

## Results

A total of 1606 patients were screened for potential eligibility in this study through the EMR. One thousand three hundred seventy-seven were ineligible and excluded. The most frequently recorded reason for patient ineligibility was the diagnosis of another rheumatic disease, followed by current treatment with TNFi therapy. One hundred eighty-six were potentially eligible but not approached based on the preference of their treating physician who had prescribed another medication or who were concerned about the medication washout period for enrollment. 13 were eligible but declined study participation.

**Fig. 1:**
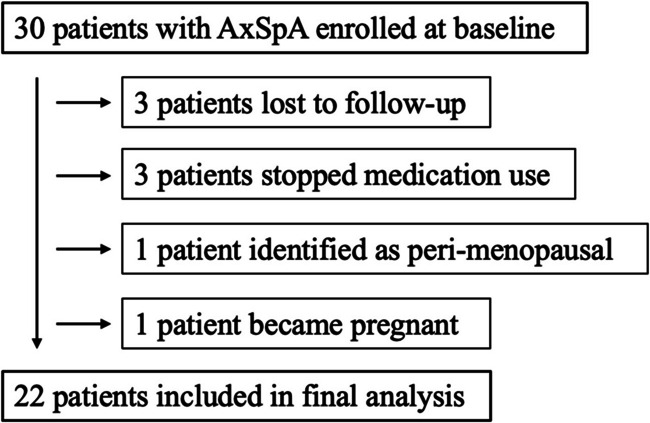
Flowchart for inclusion of study patients in final analysis

A total of 30 participants were prospectively enrolled in our study, including 17 participants with radiographic AxSpA, or AS, and 13 participants with non-radiographic AxSpA (based upon sacroiliac inflammation by MRI). 8 participants were not included in the final analysis. 6 were lost to follow-up or electively stopped medication use and 2 were found to have exclusions that were not identified at the screening visit (Fig. 1). Three patients stopped medication use due to personal preference (did not wish to make trips to medical center and risk exposure to SARS-CoV-2, or moved away from the study site during the pandemic). The cohort was predominantly White (70%). The mean age was 43 years, and 50% were female (Table 1). Approximately half of the cohort (47%) was HLA-B27 positive. Participants had moderately active disease with a mean BASDAI of 5 and a mean ASDAS-CRP of 3. Disease-related impairment of function and mobility, measured respectively as BASFI and BASMI, was numerically higher in males than females (4 vs 3; 3 vs 2, respectively). Disease duration was also longer in males than females (14 vs 9 years, respectively). More than half (57%) of the cohort reported vitamin D supplementation, and 17% reported calcium supplementation. Nearly one third (27%) reported the use of prior biologic therapy (20% of enrolled females, 33% of enrolled men). Serum inflammatory markers, ESR and CRP, were within normal limits for the majority of patients (~ 80%).

**Table 1:** Demographics of study patients

Demographic characteristics*	Overall (*N* = 30)	Study completers (*n* = 22)
Age (years, mean ± SD)	43 (14)	39 (13)
Female	15 (50%)	12 (55%)
Sex, menopausal state		
Females, premenopausal	10 (33%)	8 (36%)
Females, postmenopausal	4 (13%)	4 (18%)
Females, perimenopausal	1 (3%)	0 (0%)
Race		
White	21 (70%)	14 (64%)
Black	4 (13%)	3 (14%)
Asian	2 (7%)	2 (9%)
Other	3 (10%)	3 (14%)
Hispanic/Latino ethnicity	6 (20%)	6 (27%)
Disease characteristics		
AxSpA symptom duration (years, mean ± SD)	11.7 (10.7)	9.15 (8.68)
HLA-B27 positive	14 (47%)	10 (45%)
BASDAI (0–10) (units, mean ± SD)	5 (2)	5 (2)
BASFI (0–10) (units, mean ± SD)	4 (2)	4 (2)
BASMI (0–10) (units, mean ± SD)	3 (1)	3 (1)
m-SASSS (units, mean ± SD) **	11.7 (9.7)	10.1 (9.9), *n* = 21
Baseline CRP (mg/dL, mean ± SD)	2.24 (4.12)	0.932 (1.3)
Ankylosing Spondylitis Disease Activity Score (ASDAS)-CRP (units, mean ± SD)	3 (1)	3 (1)
Medical history		
Body Mass Index (mean ± SD)	28 (6)	28 (6)
Ever smoker	12 (40%)	7 (32%)
Areal bone mineral density		
Trabecular bone score (TBS) (mean ± SD)	1.381 (0.133)	1.384 (0.147)
Lumbar spine (Z-score, mean ± SD)	− 0.228 (1.879), *n* = 29	− 0.536 (1.630), *n* = 21
Total Hip BMD (Z-score, mean ± SD)	− 0.170 (0.907), *n* = 28	− 0.155 (0.979), *n* = 20
Femoral neck (Z-score, mean ± SD)	− 0.230 (1.012), *n* = 28	− 0.322 (1.111), *n* = 20
1/3 radius (Z-score, mean ± SD)	− 0.227 (1.163)	− 0.302 (1.111)

*HLA-B27* human leukocyte antigen B27, *BASDAI* Bath Ankylosing Spondylitis Disease Activity Index, *BASFI* Bath Ankylosing Spondylitis Functional Index, *BASMI* Bath Ankylosing Spondylitis Metrology Index, *m-SASSS* modified Stoke Ankylosing Spondylitis Spinal Score

*Values represent number and proportion (%) unless otherwise noted

** m-SASSS measurements (total score range 0–36) are calculated as 12 times the mean score of all scoring sites due to the absence of adequate cervical and lumbar x-ray films of each vertebra for each patient

***Bold values indicate a statistically significant difference between groups (*p* < 0.05)

At 12 months, 81% of participants reported 100% drug adherence. At 24 months, 53% of participants were at least 80% adherent. There was improvement in disease activity (BASDAI decreased by 33%, *p*-value = 0.003) and in function (BASMI decreased by 24%, *p*-value = 0.01) over the first 12 months of therapy, and these disease metrics remained stable from 12 to 24 months (Table 2). There were no other significant changes in disease metrics across the course of the study.

**Table 2:** Percent change from baseline in disease indices, areal bone mineral density by DXA, and bone microarchitecture by HR-pQCT in patients with AxSpA at 12 months and 24 months after IL-17 blockade treatment

	Baseline to 12 months			12 months to 24 months	
	Mean %, SD		Paired *t*-test (*p*-value)*	Mean %, SD	Paired *t*-test (*p*-value)*
Axial spondyloarthritis disease metrics					
m-SASSS	17.9% (60.3)	0.20		** − **10.3% (79.7)	0.57
BASDAI	** − 33.3%** **(44.7)**	**0.003**		7.62% (38.2)	0.40
BASFI	** − **11.4% (93.6)	0.58		** − **6.79% (73.0)	0.67
BASMI	** − 24.4%** **(41.0)**	**0.01**		** − **3.48% (25.3)	0.56
DXA (areal BMD)					
Lumbar spine	2.40% (5.86)	0.07		** − **0.334% (4.25)	0.75
1/3 Radius	** − **1.03% (4.70)	0.32		** − **0.748% (2.57)	0.24
Total hip	0.21% (2.29)	0.67		** − 1.62%** **(2.58)**	**0.02**
Femoral neck	0.21% (3.21)	0.76		** − **1.59% (3.54)	0.07
Tibia HR-pQCT					
Total vBMD	** − **1.38% (3.18)	0.06		** − **0.596% (3.20)	0.43
Cortical vBMD	** − 0.605% (1.30)**	**0.04**		** − **0.187% (1.98)	0.67
Trabecular vBMD	** − **1.627% (4.52)	0.11		** − **0.421% (4.17)	0.67
Cortical thickness	** − **0.809% (2.55)	0.16		** − **0.220% (3.85)	0.80
Cortical porosity	0.197% (13.9)	0.95		**8.27%** **(14.7)**	**0.03**
Trabecular number	** − **0.89% (4.48)	0.37		** − **1.14% (3.51)	0.17
Trabecular thickness	0.32% (2.08)	0.49		** − **0.328% (2.19)	0.52
Cortical area	** − **0.702% (2.52)	0.21		** − **0.172% (3.17)	0.81
Trabecular area	0.119% (0.678)	0.43		0.121% (0.635)	0.42
Trabecular BV/TV	** − **1.93% (4.76)	0.08		** − **1.187% (4.56)	0.27
Radius HR-pQCT					
Total vBMD	** − 1.91%** **(3.68)**	**0.03**		0.178% (2.24)	0.74
Cortical vBMD	** − **0.562% (1.71)	0.15		0.367% (1.14)	0.18
Trabecular vBMD	** − **1.83% (4.24)	0.06		0.883% (2.88)	0.21
Cortical thickness	** − **1.10% (3.34)	0.15		** − **0.839% (1.94)	0.08
Cortical porosity	75.4% (278)	0.23		** − **50.3% (314)	0.45
Trabecular number	** − **1.28% (4.92)	0.25		** − **0.667% (2.21)	0.23
Trabecular thickness	** − **0.41% (2.30)	0.43		** − **0.085% (1.60)	0.82
Cortical area	** − **1.53% (4.20)	0.11		** − **0.508% (1.99)	0.30
Trabecular area	0.382% (1.53)	0.26		0.090% (0.660)	0.58
Trabecular BV/TV	** − **2.77% (6.17)	0.051		1.34% (3.72)	0.14

*m-SASSS* modified Stoke Ankylosing Spondylitis Spinal Score, *BASDAI* Bath Ankylosing Spondylitis Disease Activity Index, *BASFI* Bath Ankylosing Spondylitis Disease Activity Index, *BASMI* Bath Ankylosing Spondylitis Metrology Index, *vBMD* volumetric bone mineral density

*Bold values indicate statistical significance (*α* = 0.05)

Mean HR-pQCT and DXA Z-scores were within 1 SD of the age- and sex-matched reference cohort at baseline, with the exception of total vBMD in men (− 1.2 SD below mean). There was little change in skeletal indices over 24 months of therapy (Table 2). Change was observed by DXA only in hip aBMD, which remained stable for the first 12 months before decreasing from 12 to 24 months (− 1.62%, *p* = 0.02). By HR-pQCT, cortical vBMD decreased over the first 12 months (− 0.61%, *p* = 0.04), and was stable from 12 to 24 months. Cortical porosity was stable over the first 12 months and then increased between 12 and 24 months of therapy (8.3%, *p* = 0.03). At the radius, the total vBMD decreased 1.9% (*p* = 0.03) over the first 12 months of therapy and then remained stable from 12 to 24 months. There were no other changes in bone measurements, and no changes were observed in a post-hoc analysis according to menopausal status. No vertebral fractures were observed at baseline, and no new vertebral fractures were observed in spine images across the 2 years of therapy. Review of patient electronic medical records prior to each study visit did not identify any new incidence of broken bones or other back or spine problems.

The biomarker panel showed little change over 24 months (Supplementary Table 2). Total alkaline phosphatase decreased by 8.74% (*p*-value = 0.06), and IL-17 levels increased, as expected per known pharmacokinetics, given the slower clearance of the larger antibody-bound IL-17 construct from the circulation in individuals being treated with secukinumab [27].

## Discussion

This data from a 2-year prospective cohort study found that participants with AxSpA with moderate disease activity who began secukinumab had improvements in clinical symptoms and function, but no change or small declines in BMD and microarchitecture and no change in bone turnover markers. There were no new vertebral fractures over the 2 years of study follow-up.

Osteoporosis with fragility fractures is a significant co-morbidity in patients with AxSpA, but the impact of therapy with IL-17 blockade on bone health has not been well explored. We carefully evaluated patients and included a comprehensive set of inclusion criteria to ensure that our cohort only included patients with active AxSpA, not fibromyalgia or other conditions that may have elevated disease activity indices. Contrary to our hypothesis that skeletal health measured by DXA, HR-pQCT, and bone biomarkers would improve with treatment, we found little evidence of improvement over 2 years of secukinumab therapy. We did observe improvement in BASDAI which, although not as large as reported in some trials, still exceeded the mean clinically important difference of 22.5% reported in the 2-year *Measure 1* clinical trial [28]. As previously mentioned, aBMD measured by DXA has recognized limitations in the setting of AxSpA due to ossification of spinal ligaments and syndesmophytes, the inability to measure trabecular and cortical bone separately, and the inability to assess microarchitecture, as all contribute to bone strength [29, 30]. This artifact may limit the ability to detect changes in aBMD with treatment in this population. Additionally, our cohort presented with baseline TBS and Z-scores in the normal range. A large meta-analysis of multiple randomized controlled trials demonstrated that BMD may still increase among patients with normal baseline BMD when treated with medications that have a favorable impact on bone [31]. However, patients initiating therapy from a more compromised state would be expected to sustain greater increases in BMD. This may have contributed to the lack of BMD increase observed across our study. HR-pQCT measurements at the tibia correlate well with bone strength at the femur and spine [32], suggesting this assessment may be useful in measuring skeletal health in AxSpA.

In a post hoc analysis of the 2-year *Measure 1* secukinumab clinical trial data, aBMD was measured by DXA at the lumbar spine and hip, and bone biomarkers were assessed. At study onset, the mean BASDAI was 6.4, 45% of patients had low BMD at the lumbar spine, and 58% had low BMD at the femoral neck. Preservation or slight improvement of BMD at the lumbar spine was seen after 2 years of secukinumab therapy, but vertebral fractures were not reported. Similar to our study, bone turnover markers were unchanged [12]. Although we observed an initial increase in total alkaline phosphatase, further examination suggested this was driven by the increase in the hepatic fraction and likely did not represent a change in bone metabolism. Other studies reported on BMD measured by DXA after TNFi therapy in patients with longstanding, moderately active AxSpA (baseline BASDAI scores of 5.4–6.0). Although BMD improved, vertebral fracture number and severity increased [14, 33, 34]. This favorable effect of TNFi therapy on BMD may highlight a more pronounced effect of TNFi therapy on osteoclast activation through the RANKL–RANK pathway, although both TNFi and IL-17 blockade therapies have been shown to decrease RANKL expression [35, 36]. A future head-to-head comparison would be necessary to comment on the effects of TNFi vs. IL-17 blockade on BMD. As reported by Deminger et al., in a TNFi-treated AxSpA cohort with low disease activity (baseline BASDAI of 2.2), no new vertebral fractures were described after 5 years of therapy [29]. In our study, there were no fractures seen over 2 years of secukinumab therapy despite moderate disease activity (baseline BASDAI of 5), although our cohort is small and underpowered to assess fracture incidence.

This study has limitations. Our sample size was small and the study was only of 2 years duration. A study of longer duration with more participants may have revealed more subtle skeletal changes. As we lacked an untreated AxSpA control group, we cannot determine whether treatment may have in fact attenuated larger changes in the bone that would have occurred as a result of the disease alone. Additionally, the heterogeneity of our cohort from the inclusion of men and postmenopausal women may have also contributed to confounding. However, the cohort was very carefully screened and potential causes of secondary bone loss were ruled out, enabling us to better isolate the effects of treatment. Our cohort also demonstrated a low prevalence of HLA-B27 positivity, but HLA-B27 positivity is less common in both Black and Latino patients [37, 38], who comprised 13% and 27% of our cohort, respectively. This likely presented a lower prevalence of HLA-B27 positivity in our cohort. While our cohort did not sustain vertebral fractures over the course of the study despite moderate disease activity, we were likely underpowered for this assessment. Finally, as most cases were enrolled and followed through the COVID-19 pandemic, normal activity such as simple walking outdoors or exercise in a gym was no doubt affected by lockdowns and other public health measures. Loading of bone through physical activity was not assessed to account for this likely effect on participant activity levels. We further faced challenges in patient adherence to medication due to frequent missed visits because of concern regarding SARS-CoV-2 exposure.

In summary, we found that despite symptomatic benefits of therapy with secukinumab, with improvements in pain and function, there were few biochemical, densitometric, or microarchitectural changes in skeletal health over two years of treatment with secukinumab. Larger, longer-term controlled studies using sensitive metrics such as HR-pQCT to follow bone quality are needed to improve our understanding of bone health in AxSpA and the relation to disease activity and therapy.

## Supplementary Information

Below is the link to the electronic supplementary material.

Supplementary file1 (DOCX 22 KB)

## Data Availability

The datasets and materials used and/or analyzed are available from the corresponding author.
