# Supplementary material for: Effects of secukinumab on skeletal microarchitecture and vertebral fractures in patients with axial spondyloarthritis using HR-pQCT
Source: Arch Osteoporos. 2025 Jun 7;20(1):73. doi: 10.1007/s11657-025-01565-w (PMC12145278; doi:10.1007/s11657-025-01565-w)
Supplement: Supplementary file 1 — Supplementary file1 (DOCX 22 KB) [file 11657_2025_1565_MOESM1_ESM.docx]

**Supplementary Table 1.** Mean disease indices, areal bone mineral density by DXA, and bone microarchitecture by HR-pQCT in patients with AxSpA at baseline, 12-months, and 24-months after IL-17 blockade treatment

|  | **Baseline** | | **12-months** | | **24-months** | |
| --- | --- | --- | --- | --- | --- | --- |
|  | N | Mean (SD) | N | Mean (SD) | N | Mean (SD) |
| Axial Spondyloarthritis disease metrics | | | | | | |
| m-SASSS | 21 | 10.13 (9.883 | 21 | 10.39 (9.294) | 19 | 11.69 (10.44) |
| BASDAI | 22 | 4.811 (1.969 | 22 | 3.191 (2.376) | 22 | 2.993 (2.277) |
| BASFI | 22 | 3.782 (2.297 | 22 | 2.755 (2.269) | 22 | 2.400 (2.107) |
| BASMI | 22 | 2.620 (1.476 | 22 | 2.073 (1.546) | 21 | 2.238 (1.681) |
| DXA (areal BMD) | | | | | | |
| Lumbar spine | 22 | 0.973 (0.182 | 21 | 1.001 (0.166) | 20 | 1.056 (0.195) |
| 1/3 Radius | 22 | 0.714 (0.082 | 21 | 0.709 (0.082) | 20 | 0.697 (0.075) |
| Total hip | 22 | 0.938 (0.132 | 20 | 0.934 (0.120) | 19 | 0.943 (0.139) |
| Femoral neck | 22 | 0.809 (0.142 | 20 | 0.802 (0.127) | 19 | 0.815 (0.161) |
| Tibia HR-pQCT | | | | | | |
| Total vBMD | 22 | 296.3 (51.34) | 21 | 288.4 (48.10) | 20 | 273.8 (45.26) |
| Cortical vBMD | 22 | 914.3 (73.01) | 21 | 903.4 (69.60) | 20 | 883.0 (74.66) |
| Trabecular vBMD | 22 | 159.4  (37.7) | 21 | 157.0 (37.83) | 20 | 150.21 (37.87) |
| Cortical thickness | 22 | 1.479 (0.226) | 21 | 1.454 (0.215) | 20 | 1.410 (0.208) |
| Cortical porosity | 22 | 0.023 (0.013) | 21 | 0.023 (0.012) | 20 | 0.029 (0.014) |
| Trabecular number | 22 | 1.365 (0.220) | 21 | 1.353 (0.216) | 20 | 1.322 (0.231) |
| Trabecular thickness | 22 | 0.245 (0.022) | 21 | 0.246 (0.023) | 20 | 0.243 (0.022) |
| Cortical area | 22 | 136.5 (28.74) | 2 | 135.8 (28.13) | 20 | 133.7 (27.22) |
| Trabecular area | 22 | 641.7 (199.9) | 21 | 655.8 (195.4) | 20 | 685.5 (214.2) |
| Trabecular BV/TV | 22 | 0.237 (0.052) | 21 | 0.233 (0.052) | 20 | 0.223 (0.052) |
| Radius HR-pQCT | | | | | | |
| Total vBMD | 22 | 315.3 (60.91) | 21 | 304.6 (56.36) | 20 | 296.8 (58.58) |
| Cortical vBMD | 22 | 909.8 (68.97) | 21 | 899.8 (67.79) | 20 | 890.3 (69.01) |
| Trabecular vBMD | 22 | 149.9 (23.75) | 21 | 147.8 (21.90) | 20 | 148.4 (23.73) |
| Cortical thickness | 22 | 1.007 (0.210) | 21 | 0.984 (0.202) | 20 | 0.951 (0.188) |
| Cortical porosity | 22 | 0.005 (0.005) | 21 | 0.009 (0.015) | 20 | 0.008 (0.006) |
| Trabecular number | 22 | 1.469 (0.200) | 21 | 1.448 (0.174) | 20 | 1.446 (0.152) |
| Trabecular thickness | 22 | 0.221 (0.012) | 21 | 0.220 (0.013) | 20 | 0.221 (0.016) |
| Cortical area | 22 | 62.43 (14.28) | 21 | 61.38 (14.31) | 20 | 60.83 (14.07) |
| Trabecular area | 22 | 240.6 (86.27) | 21 | 246.23 (85.29) | 20 | 257.9 (91.79) |
| Trabecular BV/TV | 22 | 0.214 (0.036) | 21 | 0.210 (0.034) | 20 | 0.211 (0.037) |

** Bold values indicate statistical significance (α=0.05)*

*Abbreviations include: m-SASSS, modified Stoke Ankylosing Spondylitis Spinal Score; BASDAI,*

*Bath Ankylosing Spondylitis Disease Activity Index; BASFI, Bath Ankylosing Spondylitis Disease*

*Activity Index; BASMI, Bath Ankylosing Spondylitis Metrology Index; vBMD, volumetric bone*

*mineral density*

**Supplementary Table 2**. Mean bone biomarkers and cytokine levels in patients with Axial Spondyloarthritis at baseline, 2 months, 12 months, and 24 months after IL-17 blockade treatment*

| **Biomarkers**** | **Baseline** | | **2** **months** | | **12 months** | | **24 months** | |
| --- | --- | --- | --- | --- | --- | --- | --- | --- |
|  |  |  |  |  |  |  |  |  |
|  | N | Mean (SD) | N | Mean (SD) | N | Mean (SD) | N | Mean (SD) |
| Alkaline phosphatase | 19 | 88.3 (29.4) | 8 | 85.3 (29.4) | 22 | 78.0 (27.2) | 16 | 79.9 (30.3) |
| Bone specific alkaline phosphatase | 18 | 39.2 (11.9) | 8 | 37.5 (18.5) | 21 | 35.9 (11.9) | 15 | 36.9 (16.0) |
| Liver specific alkaline phosphatase | 17 | 52.1 (27.9) | 8 | 47.8 (16.1) | 21 | 42.4 (19.6) | 15 | 44.5 (19.5) |
| iPTH | 22 | 23.0 (9.17) |  |  |  |  |  |  |
| Anti-TNFa | 22 | 2.93 (3.55) | 15 | 2.52 (2.83) | 22 | 5.64 (10.4) | 16 | 6.10 (13.1) |
| CTX | 22 | 472  (167) | 15 | 458  (201) | 22 | 401  (132) | 16 | 473  (199) |
| IL-1B | 22 | 6.50 (0.00) | 15 | 6.50 (0.00) | 22 | 6.50 (0.00) | 16 | 6.50 (0.00) |
| Osteocalcin | 22 | 19.7 (6.03) | 15 | 20.5 (6.60) | 22 | 19.4 (6.64) | 16 | 21.5 (9.22) |
| IL-17 | 22 | 1.55 (0.68) | 15 | 3.47 (3.80) | 22 | 2.60 (2.67) | 16 | 2.56 (2.29) |

** Bold values indicate statistical significance (α=0.10)*

*** All biomarker values are reported as mean (standard deviation) unless otherwise noted. iPTH levels measured only at baseline*

*Abbreviations include: iPTH, intact parathyroid hormone; anti-TNFa, anti-tumor necrosis factor alpha; CTX, c-terminal telopeptide; IL-1B, interleukin-1B; IL-17, interleukin-17*
